# Source Apportionment and Health Risk Assessment of PM_2.5_-Bound Heavy Metals in Wintertime Laibin, Guangxi, China

**DOI:** 10.3390/toxics14100923

**Published:** 2026-10-19

**Authors:** Rui Qu, Jingying Mao, Jian Gao, Kuiquan Duan, Yuanguan Gao, Haisheng Li, Chuan Nong, Jiahui Lu, Hexiao Chen

**Affiliations:** 1 State Key Laboratory of Environmental Criteria and Risk Assessment, Chinese Research Academy of Environmental Sciences, Beijing 100012, China; qurui@craes.org.cn (R.Q.); duankuiquan@craes.org.cn (K.D.); gaoyg@craes.org.cn (Y.G.); 2 Guangxi Research Academy of Environmental Sciences, Nanning 530022, China; 13978185061@163.com (J.M.); nongchuan@gxhky.cn (C.N.); lujiahui@gxhky.cn (J.L.); chenhexiao@gxhky.cn (H.C.)

**Keywords:** PM_2.5_, heavy metals, sources, health risk assessment

## Abstract

PM_2.5_-bound heavy metals are of concern because of their persistence, toxicity, and inhalation-related carcinogenic and noncarcinogenic effects. Concentrations, emission sources, and inhalation health risks of these pollutants were investigated during a winter campaign lasting 42 days in 2025 in Laibin, China. The mean PM_2.5_ concentration was 47.6 μg/m^3^, with nominally higher nighttime levels associated with lower wind speeds and a shallower nocturnal boundary layer. The total concentration of nine measured heavy metals (Zn, Cr, Ni, Pb, V, Cu, Sb, Cd, and Co) averaged 363.0 ng/m^3^, dominated by Zn, Cr, and Ni. Enrichment factors indicated severe Cd enrichment, significant Ni enrichment, and moderate enrichment of V, Cu, Cr, Pb, and Zn, suggesting anthropogenic influence, especially for Cd and Ni. PMF analysis preliminarily resolved three major sources in the daily run: mixed non-ferrous metal smelting and coal combustion source (43%), traffic (23%), and industrial combustion (34%). In the separate daytime and nighttime runs, industrial combustion was the largest source in both periods, whereas traffic increased at night. Non-carcinogenic hazard index (HI) values were below 1 for all populations (children > adult males > adult females), and carcinogenic risks (CR) slightly exceeded 1 × 10^−4^ under winter exposure scenarios, with Cr as the main contributor. These results provide a winter-only baseline; precautionary Cr controls are suggested for winter pollution episodes, and multi-season monitoring is needed for comprehensive air quality strategies.

## 1. Introduction

PM_2.5_ is commonly defined as airborne particulate matter with an aerodynamic diameter of 2.5 μm or less, which, driven by rapid urbanization and industrialization, has become a widespread and severe air pollutant globally [1,2]. Such particles not only affect atmospheric visibility and regional climate but also pose a significant threat to human health [3,4]. Their small diameter and large surface area enable PM_2.5_ to adsorb a wide range of hazardous materials, including heavy metals and organic compounds [5]. Heavy metals, as important components of PM_2.5_, are non-biodegradable, highly accumulative, acutely toxic, and characterized by long atmospheric residence times; human exposure occurs through respiratory intake, dietary ingestion, and dermal absorption, potentially leading to various adverse health outcomes [6]. Several PM_2.5_-bound metals are of particular health concern because their toxicity and carcinogenicity depend strongly on chemical form and oxidation state. For example, Cr(VI) compounds and nickel compounds have been classified as carcinogenic to humans by the International Agency for Research on Cancer (IARC), whereas the evidence differs for metallic Cr, Cr(III), and metallic Ni [7,8]. For example, health risk assessments in Agra, India, identified Cr, Cd, and As as the major contributors to carcinogenic risk and Mn and Cr as the main contributors to non-carcinogenic risk [9], while in Ilorin, Nigeria, PM_2.5_-bound toxic metals exceeded acceptable carcinogenic and non-carcinogenic risk thresholds [10]. In addition, certain redox-active metals (e.g., Cr, Cu, Mn, and Zn) can interfere with the antioxidant defense system, induce excessive reactive oxygen species (ROS) generation, and consequently trigger oxidative stress and inflammatory cascades [11,12]. Beyond health impacts, heavy metals may also enter terrestrial and aquatic ecosystems through atmospheric deposition, causing ecological damage [13,14,15].

Emission source compositions, economic structures, and meteorological conditions vary considerably across regions, resulting in pronounced spatial heterogeneity in the source characteristics of heavy metals in PM_2.5_ [16,17]. Such heterogeneity requires source apportionment specific to each region for differentiated emission reduction. Traffic, industrial emissions, and fuel combustion dominate PM_2.5_-bound heavy metals in Europe and North America [18,19,20], while traffic-related emissions and combustion sources prevail in Bangkok, Southeast Asia [4]. These contrasts indicate that source contributions vary by region and that apportionment results are not directly transferable. Source apportionment is further complicated by the fact that the majority of heavy metals in the atmosphere are products of multiple emission sources mixed under specific meteorological conditions, which introduces substantial uncertainty into source tracing [21]. To reduce this uncertainty, diagnostic elemental ratios and tracer species have been widely employed to identify specific sources. Several heavy metal elements have been confirmed as effective tracers, such as antimony (Sb) for brake wear [22], Cd for high-temperature volatilization of lubricating oils and non-exhaust emissions from vehicles [23,24], and the combination of vanadium (V) and nickel (Ni) for fuel combustion and high-temperature industrial processes [25]. These fingerprint signatures provide a chemical basis for source identification using receptor models.

In China, existing aerosol studies have largely concentrated on megacities such as Guangzhou, Beijing, and Tianjin [6,26,27], whereas much less attention has been paid to small- and medium-sized cities like Laibin. Laibin, situated in the central part of Guangxi Zhuang Autonomous Region, is known as the “Sugar Capital of China” for its sugarcane cultivation [28] and is also rich in coal, manganese, and other mineral resources, with widespread industrial and mining enterprises [29]. However, the sources and health risks of heavy metals bound to PM_2.5_ in Laibin remain unclear, hindering the identification of relevant pollution sources and constraining the precise formulation of regional air pollution control strategies. In view of this, this study was conducted to (1) examine the wintertime levels and temporal variations of PM_2.5_ and its heavy metal constituents in Laibin; (2) identify the primary emission sources using enrichment factors and the PMF receptor model; and (3) quantify the cancer and non-cancer risks from these metals, with the aim of providing a scientific reference for seasonal pollution mitigation in the city.

## 2. Materials and Methods

### 2.1. Site Descriptions, Sample Collection and Chemical Analysis

**Figure 1:**
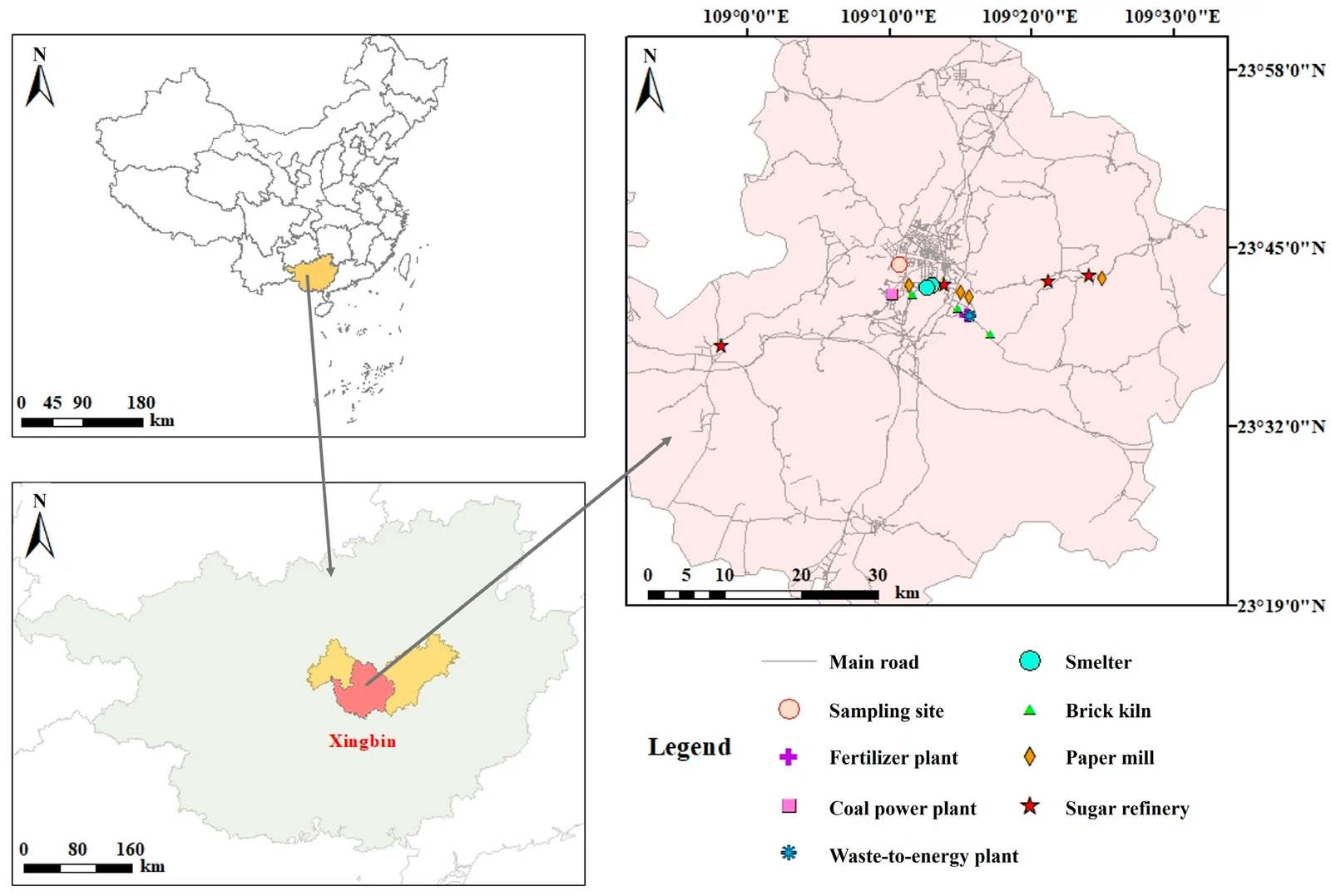
Location of the PM_2.5_ sampling site and major potential emission sources in the study area.

In this study, the sampling site was located at the People’s Government of Xingbin District (109.18° E, 23.73° N) (Figure 1). The area surrounding the sampling site is characterized by dense road networks and multiple industrial facilities, including three brick kilns, one coal-fired power plant, one fertilizer plant, four paper mills, two metal smelters, four sugar refineries, and one waste-to-energy incineration plant, which together constitute the major potential pollution sources in the study area.

All quartz fiber filters (90 mm, Pall Life Sciences, Ann Arbor, MI, USA) were preconditioned prior to use by wrapping in aluminum foil and heating at 500 °C for 4 h in a muffle furnace to eliminate organic residues, following the protocol of Li et al. (2019) [8]. Before and after sampling, each filter was weighed using a microbalance (Mettler Toledo, Columbus, OH, USA) to determine PM_2.5_ mass. Active air sampling was conducted using an L16 air sampler (Qingdao Lvlan Environmental Protection Technology Co., Ltd., Qingdao, China) at an average flow rate of 971 L/h (approximately 16.2 L/min). The air volume for each sample of about 11.3 h was calculated as flow rate × sampling duration, yielding approximately 11.0 m^3^ per sample. PM_2.5_ mass concentration was calculated as the difference between the filter weights before and after sampling divided by the corresponding air volume. The PM_2.5_ sampling campaign was conducted over the winter period from 17 January to 27 February 2025, covering approximately 42 calendar days. Each sampling day was divided into two intervals of about 11.3 h: a daytime session (06:00–18:00) and a nighttime session (18:00–06:00). Paired daytime and nighttime samples were collected during the campaign. After excluding damaged samples, a total of 80 valid PM_2.5_ filter samples were obtained, comprising 40 daytime and 40 nighttime samples. All filters were immediately stored at −20 °C until further analysis in the laboratory.

For digestion pretreatment, one-quarter of each filter was placed into a digestion vessel with HNO_3_, H_2_O_2_, and HF (6:2:0.6, *v*/*v*/*v*), and digested in a microwave digestion system (MARS, CEM Corporation, Matthews, NC, USA) following a programmed temperature procedure. The digestate was transferred to a PP bottle and diluted to 100 mL with ultrapure water (18.2 MΩ·cm). The concentrations of V, Cr, Co, Ni, Cu, Zn, Cd, Sb, Pb, and Mn were determined by inductively coupled plasma mass spectrometry (ICP-MS, ELAN DRC-e, PerkinElmer Inc., Waltham, MA, USA) [8,30]. The atmospheric concentrations of these elements (ng/m^3^) were calculated from the measured solution concentrations, the digestion volume, the filter aliquot fraction (one-quarter), and the corresponding air volume. The method detection limits (ng/m^3^) for the target elements were as follows: V 0.1, Cr 1, Co 0.03, Ni 0.5, Cu 0.7, Zn 3, Cd 0.03, Sb 0.09, Pb 0.6, and Mn 0.3. In this study, Mn was used solely as a reference element for enrichment factor calculations and was not included in the list of target heavy metals for source apportionment or health risk assessment.

### 2.2. Data Analysis

#### 2.2.1. Enrichment Factor (EF)

Enrichment factor (EF) analysis serves as a valuable metric for evaluating the extent of human-induced modification of environmental media [31]. By comparing the concentrations of heavy metal elements in atmospheric PM_2.5_ with their corresponding soil background values, the contribution of human activities to heavy metal sources can be effectively assessed. The *EF* value is calculated using the following formula:

(1) $$\mathrm{EF}=\frac{{\left(U_{\alpha }/U_{\beta }\right)}_{\mathrm{Sample}}}{{\left(U_{\alpha }/U_{\beta }\right)}_{\mathrm{Background}}}$$

where (*U_α_*/*U_β_*)_Sample_ and (*U_α_*/*U_β_*)_Background_ are the concentration ratios of the target element *U_α_* to the reference element *U_β_* in PM_2.5_ samples and in the soil background, respectively. In this study, Mn was chosen as the reference element due to its high crustal abundance and low anthropogenic interference [32]. The soil background heavy metal concentrations for Guangxi topsoil were obtained from the Chinese Environmental Background Value Database [33].

#### 2.2.2. PMF Model

PMF is a multivariate receptor model originally proposed and developed by Paatero and Tapper [34]. The fundamental equation of the PMF model can be expressed as:

(2) $$X_{\mathrm{mn}}=\sum_{\alpha =1}^{\beta }G_{\mathrm{m\beta}}F_{\mathrm{n\alpha}}+E_{\alpha \beta }$$

where *X_mn_* denotes the data matrix capturing the presence of heavy metal *n* for sample *m*; *G_mα_* represents the contribution matrix of source *α* to sample *m*; and *F_αn_* refers to the factor profile of heavy metal *n* associated with source factor *α*.

Measurement uncertainties were calculated according to the measured concentrations and method detection limits (Supplementary Equations (S1)–(S6)) following the EPA PMF 5.0 framework. The resolved factors were subsequently interpreted by combining their elemental profiles with established source tracers, inter-element correlations, and information on potential local emission sources. Detailed mathematical descriptions of the PMF algorithm are available in the EPA PMF 5.0 User Guide and related methodological literature [35,36].

#### 2.2.3. Health Risk Assessment (HRA)

The health risks arising from exposure to PM_2.5_-associated heavy metals were assessed following the health risk assessment (HRA) framework established by the U.S. Environmental Protection Agency (U.S. EPA). The target population was categorized into three groups: children, adult males, and adult females. Although PM_2.5_-bound heavy metals may be absorbed through breathing, eating, or skin contact, this analysis primarily focused on the inhalation pathway, as it constitutes the most significant exposure route [37,38]. Hazard quotients (HQ) for non-carcinogenic effects were derived for Zn, Cr, Ni, Pb, V, Cu, Cd, and Co, while carcinogenic risks (CR) were computed for Cr (VI), Ni, Pb, Cd, and Co. The Cr (VI) fraction was taken to be one-seventh of the total Cr burden [39]. The relevant formulas are presented below:

(3) $${\mathrm{ADD}}_{\mathrm{inh}}=\frac{V_{m}*\mathrm{InhR}*\mathrm{EF}*\mathrm{ED}}{\mathrm{AT}*\mathrm{BW}}$$

*CR* = *LADD_inh_* ∗ *SF* (4)

(5) $$\mathrm{HQ}=\frac{{\mathrm{ADD}}_{\mathrm{inh}}}{\mathrm{RfD}}$$

(6) $$\mathrm{HI}=\sum {\mathrm{HQ}}_{m}$$

In these equations, *ADD_inh_* refers to the average daily intake dose; *V_m_* is the concentration of metal *i*; *InhR* indicates the inhalation rate; *EF* denotes exposure frequency; *ED* signifies exposure duration; *AT* corresponds to averaging time; *BW* represents body weight; and *CR* is the carcinogenic risk via inhalation. *LADD_inh_*, the lifetime average daily dose, is distinguished from *ADD_inh_* by the use of different *AT* and *ED* values. *SF* is the slope factor; *HQ* is the hazard quotient for non-carcinogenic risk via inhalation; *RfD* is the corresponding reference dose; *HI* is the sum of *HQ*s for all eight metals (m = 8). If *HQ* or *HI* < 1, no non-carcinogenic effects are expected, whereas *HQ* or *HI* ≥ 1 suggests potential health concerns. For carcinogenic risk, in this study we adopted the widely used U.S. EPA criteria, where *CR* > 1 × 10 ^−4^ indicates a significant risk that should be prioritized for mitigation, while 1 × 10 ^−6^ < *CR* ≤ 1 × 10 ^−4^ is considered acceptable or tolerable [40].

Table 1 presents the exposure parameters, which were drawn from the China Exposure Factors Handbook (Ministry of Ecology and Environment of the People’s Republic of China, 2013). The corresponding *RfD* (mg/(kg·day)) and *SF* (mg/(kg·day)) ^−1^ values—for non-carcinogenic and carcinogenic metals, respectively—are given in Table 2 [39,41]. These are potential inhaled doses; the exhaled/undeposited fraction and post-deposition metal bioaccessibility were not quantified, so risks are conservative screening estimates.

## 3. Results and Discussion

### 3.1. PM_2.5_ Mass Concentration and Its Relationship with Environmental Factors

The wintertime PM_2.5_ mass concentration distribution in the study area is presented in Table S1. During the observation period, the daily average PM_2.5_ concentration ranged from 6.8 to 142.5 μg/m^3^, with a campaign mean of 47.6 μg/m^3^. For descriptive context, the Grade I and Grade II 24 h PM_2.5_ limits under GB 3095–2012, which was in force during the 2025 sampling campaign, were 35 and 75 μg m^−3^, respectively [42]. It also exceeds the World Health Organization (WHO) 2021 24 h guideline of 15 μg/m^3^ [43], the United States Environmental Protection Agency (U.S. EPA) 24 h standard of 35 μg/m^3^ [44], and the European Union (EU) 2030 24 h limit of 25 μg/m^3^ [45]. When compared with wintertime PM_2.5_ concentrations in other domestic cities, the concentration in the study area was lower than those in Chongqing (97.1 μg/m^3^) [14], Huangshi (71.1 μg/m^3^) [16], Fuzhou (59.8 μg/m^3^) [1], Suzhou (57.0 μg/m^3^) [46], and Nanning (65.0 μg/m^3^) [47], indicating relatively minor PM_2.5_ pollution in the study area compared with these domestic cities, although it still exceeds the WHO, U.S. EPA, and EU reference values.

**Figure 2:**
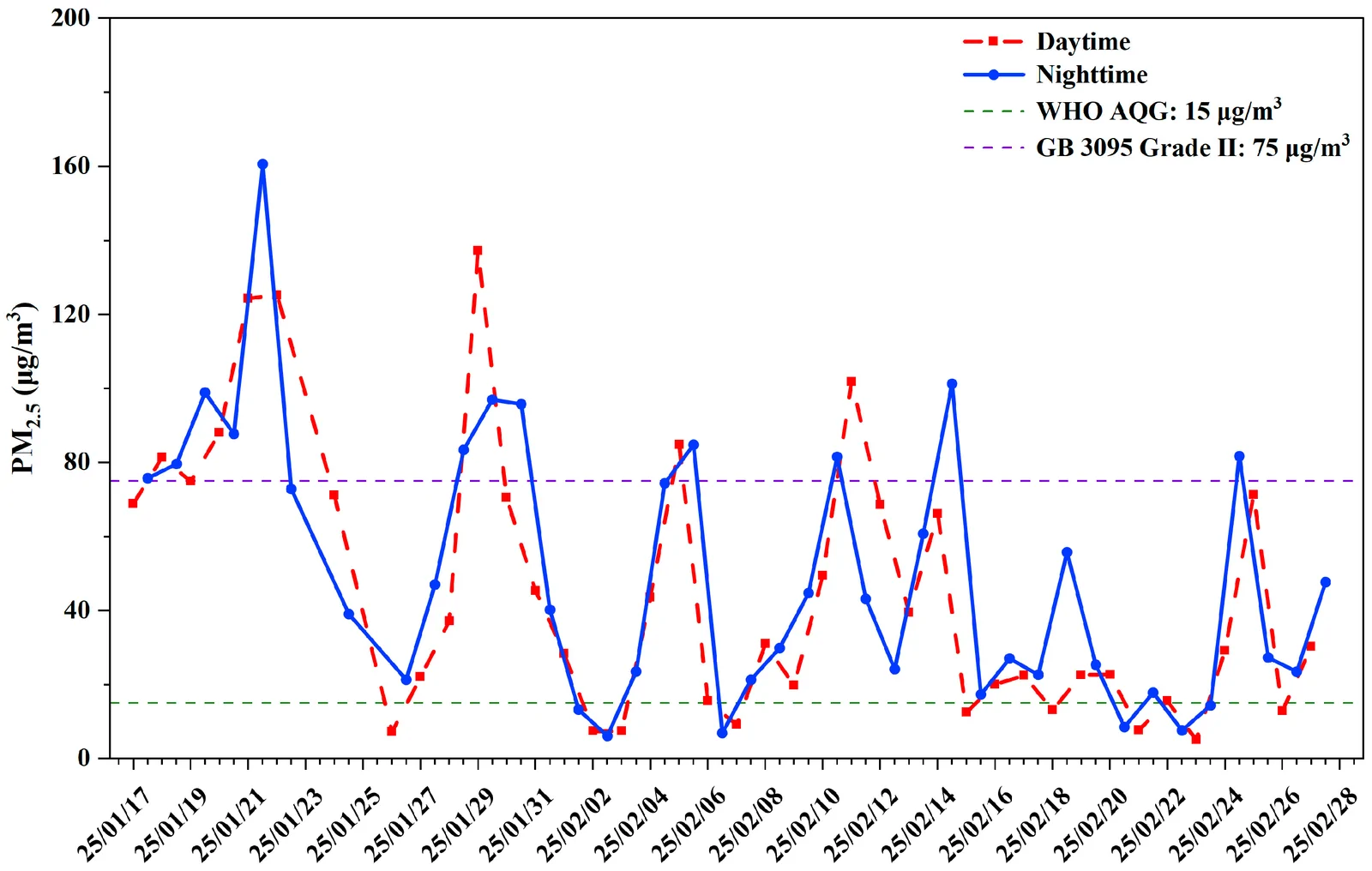
Time series of daytime and nighttime PM_2.5_ concentrations from 17 January to 27 February 2025. Daytime and nighttime samples were collected within 12 h time windows (06:00–18:00 and 18:00–06:00, respectively), with an effective sampling duration of 11.3 h for each sample. Dashed lines represent two air quality reference thresholds: WHO 2021 AQG (15 μg/m^3^) [43] and GB 3095–2012 Grade II (75 μg/m^3^) [42].

**Figure 3:**
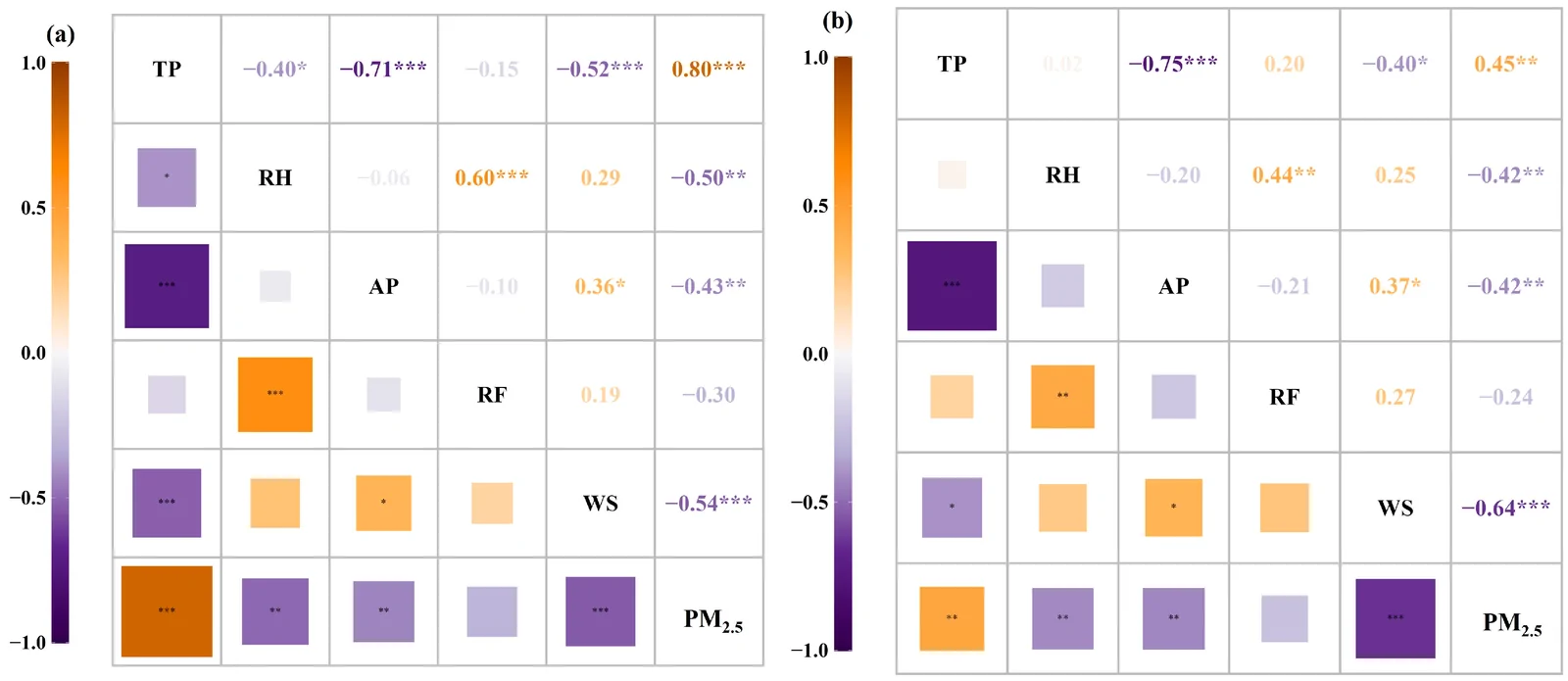
Pearson correlations between PM_2.5_ and meteorological parameters during (**a**) daytime and (**b**) nighttime. TP, temperature; RH, relative humidity; AP, atmospheric pressure; RF, rainfall; WS, wind speed. *, ** and *** denote *p* < 0.05, *p* < 0.01, and *p* < 0.001, respectively.

On average, nighttime PM_2.5_ concentrations (49.8 μg/m^3^) were slightly higher than daytime concentrations (45.3 μg/m^3^) (Figure 2), although this difference did not reach statistical significance (Wilcoxon signed-rank test, *p* = 0.154, Table S3), consistent with previous studies [48]. Meteorological conditions have been widely recognized to exert a significant influence on PM_2.5_ concentrations [49]. In the study area, both daytime and nighttime PM_2.5_ concentrations were positively correlated with temperature and negatively correlated with relative humidity, atmospheric pressure, and wind speed, although the strengths of the correlation coefficients differed between daytime and nighttime (Figure 3). During the daytime, PM_2.5_ concentration was primarily influenced by temperature, followed by wind speed, relative humidity, and atmospheric pressure. At night, the ranking of correlation strengths between meteorological factors and PM_2.5_ concentration was: wind speed > temperature > relative humidity ≈ atmospheric pressure, and it was indicated that nighttime PM_2.5_ concentration was mainly controlled by wind speed, with temperature as the secondary factor, while relatively weaker influences were exerted by relative humidity and atmospheric pressure. Regardless of daytime or nighttime, no significant correlation was shown between PM_2.5_ concentration and rainfall, which may be attributable to the scarce winter precipitation, during which the scavenging effect of rainfall on PM_2.5_ is extremely low [50].

The cumulative effect of stagnant meteorological conditions, rather than a direct chemical driving force of temperature, was reflected by the positive correlation between daytime PM_2.5_ and air temperature [51,52]. Under stagnant conditions, vertical mixing is suppressed while surface warming occurs; pollutant dispersion is further reduced by weak horizontal winds and low pressure gradients [53]. This physical interpretation was corroborated by the negative correlations of PM_2.5_ with wind speed and atmospheric pressure [54]. Nighttime wind speed exerts a stronger influence on PM_2.5_ concentrations than temperature does. This stronger influence can be attributed to the combined effects of the shrinking stable boundary layer and horizontal mechanical diffusion [55]. At night, the boundary layer shrinks, and pollutants are compressed into a shallower surface layer. For example, the boundary layer height is approximately 200 m at night versus 1200 m during the day in the Pearl River Delta region [56]. This shallow nocturnal boundary layer makes local pollutant concentrations more sensitive to horizontal transport. The negative correlation between PM_2.5_ and relative humidity (mean relative humidity ~70%) can be attributed to wind speed variability and enhanced wet scavenging under high-humidity conditions, where wet removal dominates over the hygroscopic growth effect of submicron particles [4,57].

### 3.2. Concentrations of PM_2.5_-Bound Heavy Metals

During the sampling period, the total concentration of the nine heavy metals (Zn, Cr, Ni, Pb, V, Cu, Sb, Cd, and Co) ranged from 175.6 to 857.9 ng/m^3^, with a mean value of 363.0 ng/m^3^ (Table S1). The daily average concentrations were found to follow the order: Zn (124.9 ng/m^3^) > Cr (70.6 ng/m^3^) > Ni (64.5 ng/m^3^) > Pb (37.2 ng/m^3^) > V (32.1 ng/m^3^) > Cu (27.8 ng/m^3^) > Sb (3.7 ng/m^3^) > Cd (1.6 ng/m^3^) > Co (0.7 ng/m^3^). To further contextualize these levels, wintertime observations from Chongqing [14], Huangshi [16], Fuzhou [1], and Suzhou [46]—all southern cities—were selected for comparison (Table S2). The results showed that the concentrations of Zn, Pb, Cu, and other elements in the study area exhibited notable regional fluctuations depending on the reference city (e.g., higher than those in Chongqing and Suzhou but lower than those in Huangshi and Fuzhou), without showing a consistent trend of either enrichment or depletion. In contrast, the Cr and Ni concentrations in Laibin were consistently higher than the values reported for all four comparison cities. This consistently elevated pattern suggests that Laibin may receive substantial contributions from local or regional industrial activities associated with Cr and Ni emissions, such as metallurgy, alloy production, or other high-temperature industrial processes [8,37].

**Figure 4:**
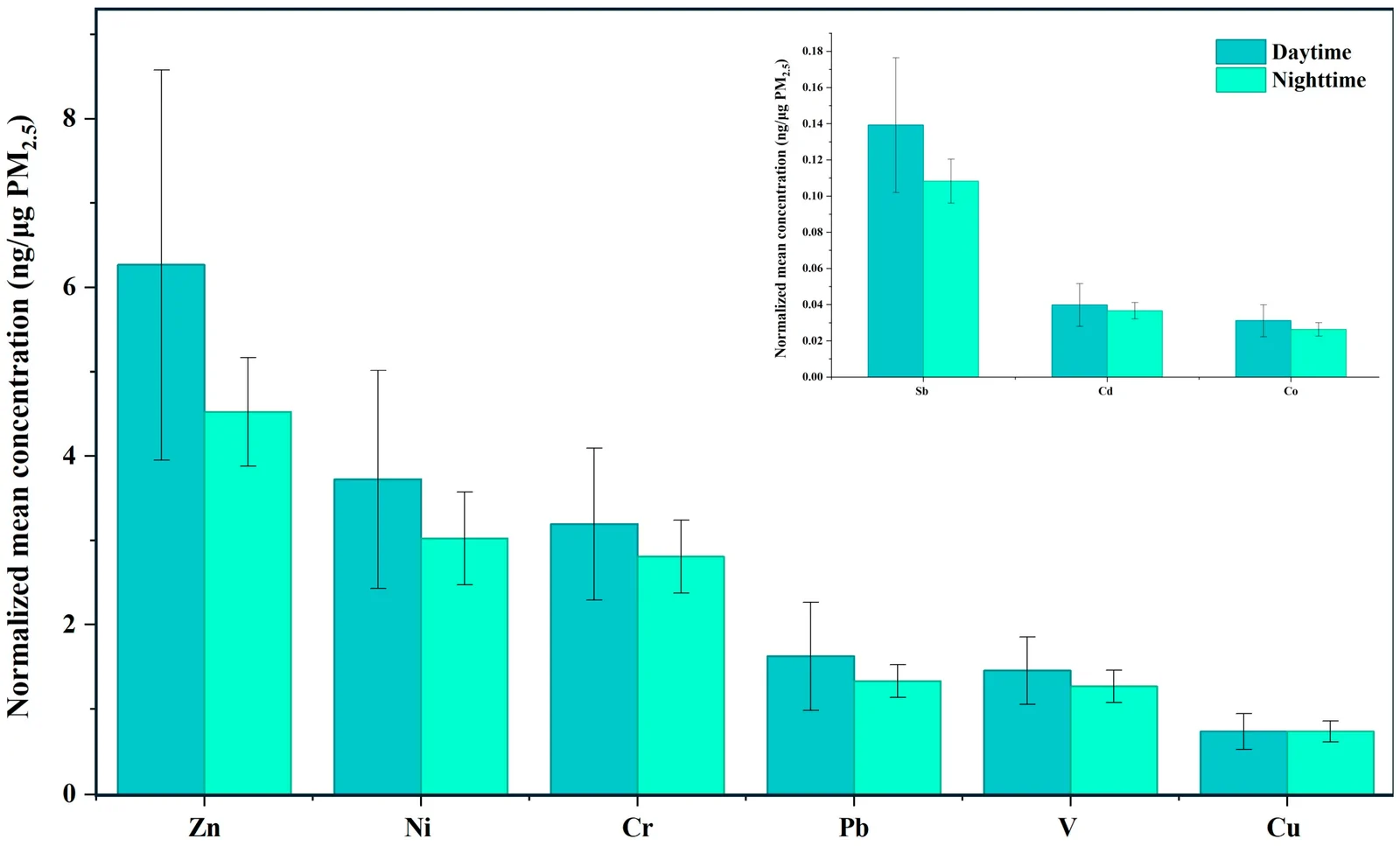
Daytime and nighttime PM_2.5_-normalized heavy metal concentrations (mean of individual metal/PM_2.5_ ratios ± SEM, *n* = 40 per group). No day–night difference was statistically significant for any metal (paired Wilcoxon signed-rank test, all *p* > 0.20; Table S3).

To further characterize the temporal variation, the day–night variations of the nine elements were analyzed separately. The total concentration of the nine elements rose from 314.3 ng/m^3^ in the daytime to 411.8 ng/m^3^ at night (a 31% increase), likely due to the reduced planetary boundary layer height and weakened atmospheric diffusion, which promote near-surface pollutant accumulation [58]. Paired Wilcoxon signed-rank tests confirmed that eight of the nine metals were significantly higher at night volumetrically (*p* < 0.05; Table S3), with only Pb non-significant (*p* = 0.116). After normalization to PM_2.5_ mass using the mean of ratios, all nine elements exhibited higher or comparable mass-normalized concentrations in the daytime (Figure 4): Zn showed the largest day–night difference, with daytime concentrations 40% higher than nighttime concentrations, whereas the mean Cu/PM_2.5_ ratio was essentially identical between day and night. However, none of these normalized differences were significant (all *p* > 0.20; Table S3). Volumetrically, however, Cu showed the greatest nighttime increase (+56%, *p* = 0.007).

The near-constant Cu/PM_2.5_ ratio indicates that the nocturnal increase in volumetric Cu reflects PM_2.5_ mass accumulation rather than additional brake-wear particle emissions. Both Cu (*p* = 0.21) and Sb (*p* = 0.47), widely used brake-wear tracers [59,60], showed no significant day–night difference after PM_2.5_ normalization. Nighttime freight traffic may contribute to the PM_2.5_ mass buildup, but the mean-of-ratios results do not support relative enrichment of Cu and Sb within nighttime PM_2.5_; notably, the ratio-of-means estimate for Cu suggested the opposite [19], underscoring that the conclusion depends on the averaging metric.

### 3.3. Enrichment Factor Analysis of PM_2.5_-Bound Heavy Metals

**Figure 5:**
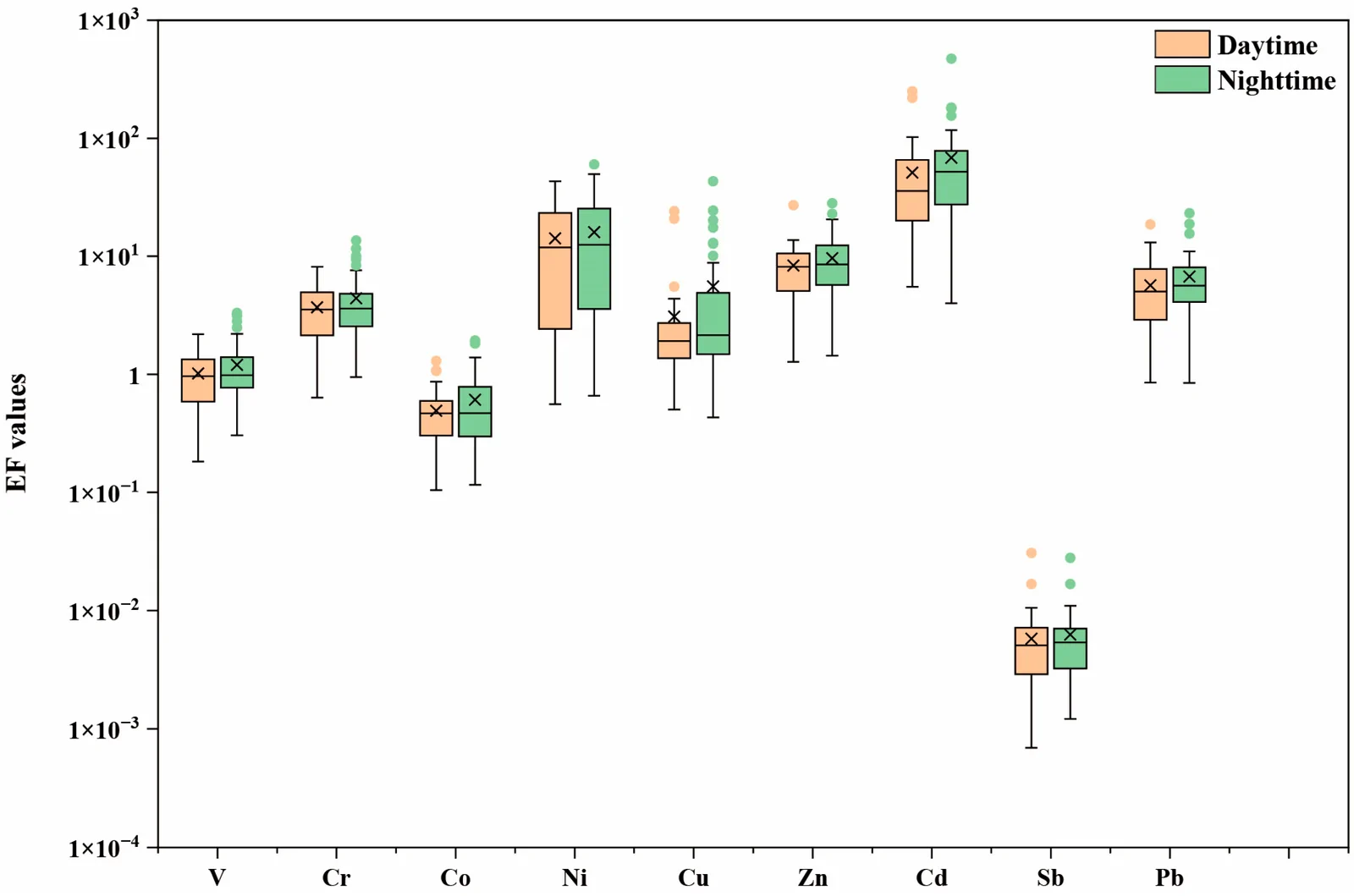
Distributions of enrichment factors (EFs) for PM_2.5_-bound heavy metals during daytime and nighttime sampling (note the logarithmic y-axis). In each box, the horizontal line denotes the median and the cross (×) indicates the mean; boxes represent the 25th–75th percentile, whiskers extend to 1.5× the interquartile range, and points beyond whiskers are outliers.

To evaluate the influence of anthropogenic emissions on heavy metals in PM_2.5_, enrichment factors (EFs) were calculated for daytime and nighttime samples (Figure 5). The EF values were computed using Mn as the reference element and Guangxi soil background values. The average *EF* values of Co and Sb were below 1 during both periods, indicating no apparent enrichment relative to the local soil background. However, low bulk *EF* values do not necessarily exclude contributions from specific anthropogenic sources, particularly when source contributions are evaluated using temporal covariance in the PMF analysis. The *EF* values of V, Cu, Cr, Pb, and Zn ranged from 1 to 10, indicating moderate enrichment and contributions from both natural and anthropogenic sources. The *EF* values of Ni were 14.2 (daytime) and 16.1 (nighttime), falling between 10 and 20, indicating significant enrichment and mainly from human activities. The *EF* values of Cd were 51.1 (daytime) and 68.4 (nighttime), far exceeding 20, indicating severe anthropogenic contamination, which may be related to industrial and mining operations in Laibin City.

### 3.4. Source Apportionment of PM_2.5_-Bound Heavy Metals

**Figure 6:**
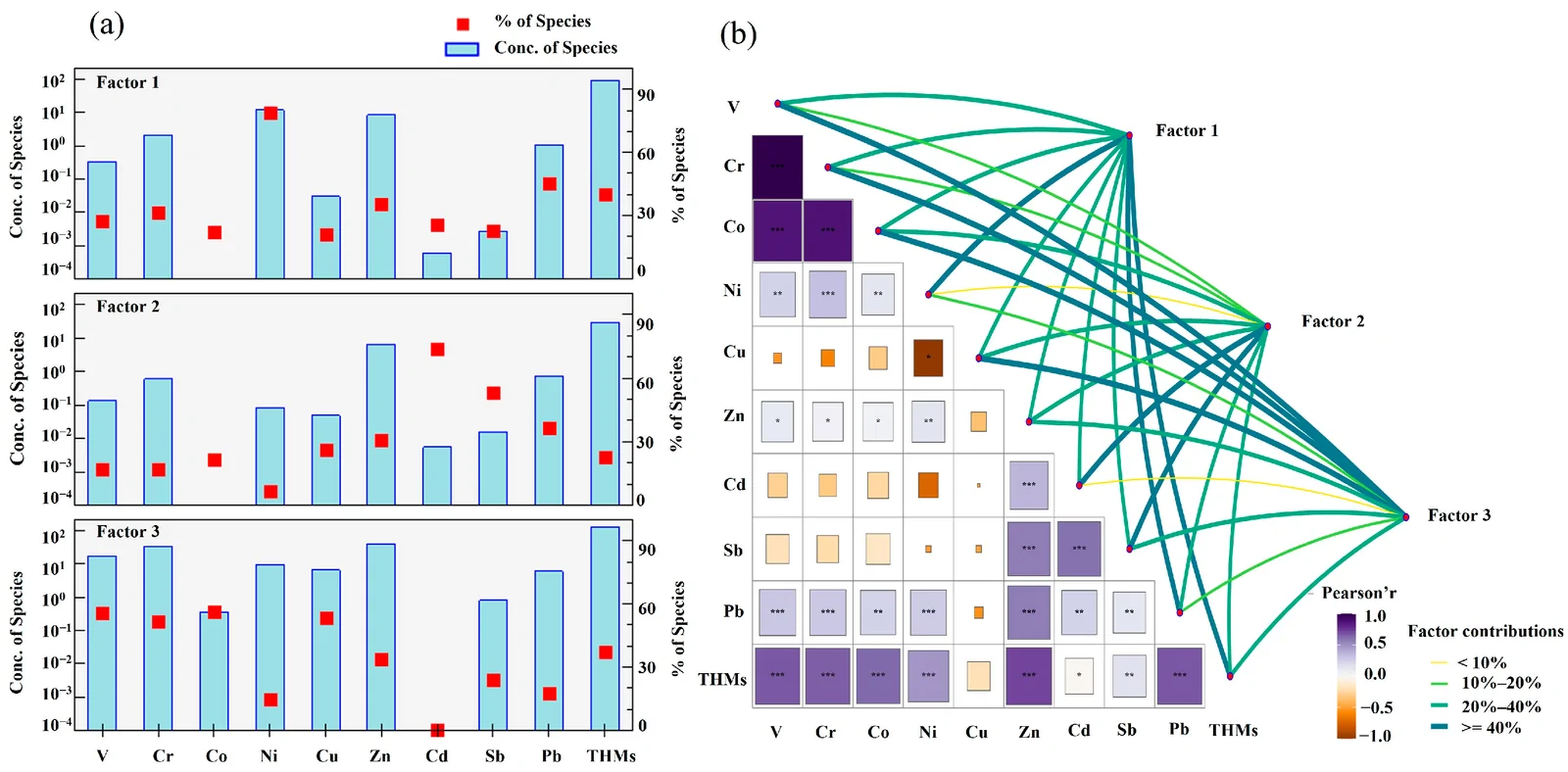
Source apportionment of heavy metals in ambient PM_2.5_. (**a**) PMF factor profiles. Blue bars: species concentrations; red squares: mass fractions of heavy metals in each factor. (**b**) Composite plot. Left heatmap: pairwise correlations among heavy metals; *, **, and *** denote statistical significance at *p* < 0.05, *p* < 0.01, and *p* < 0.001, respectively. Colored lines link each metal to its PMF factor; line color indicates the fraction of species explained: yellow < 10%, green 10–20%, teal 20–40%, dark blue ≥ 40%.

**Figure 7:**
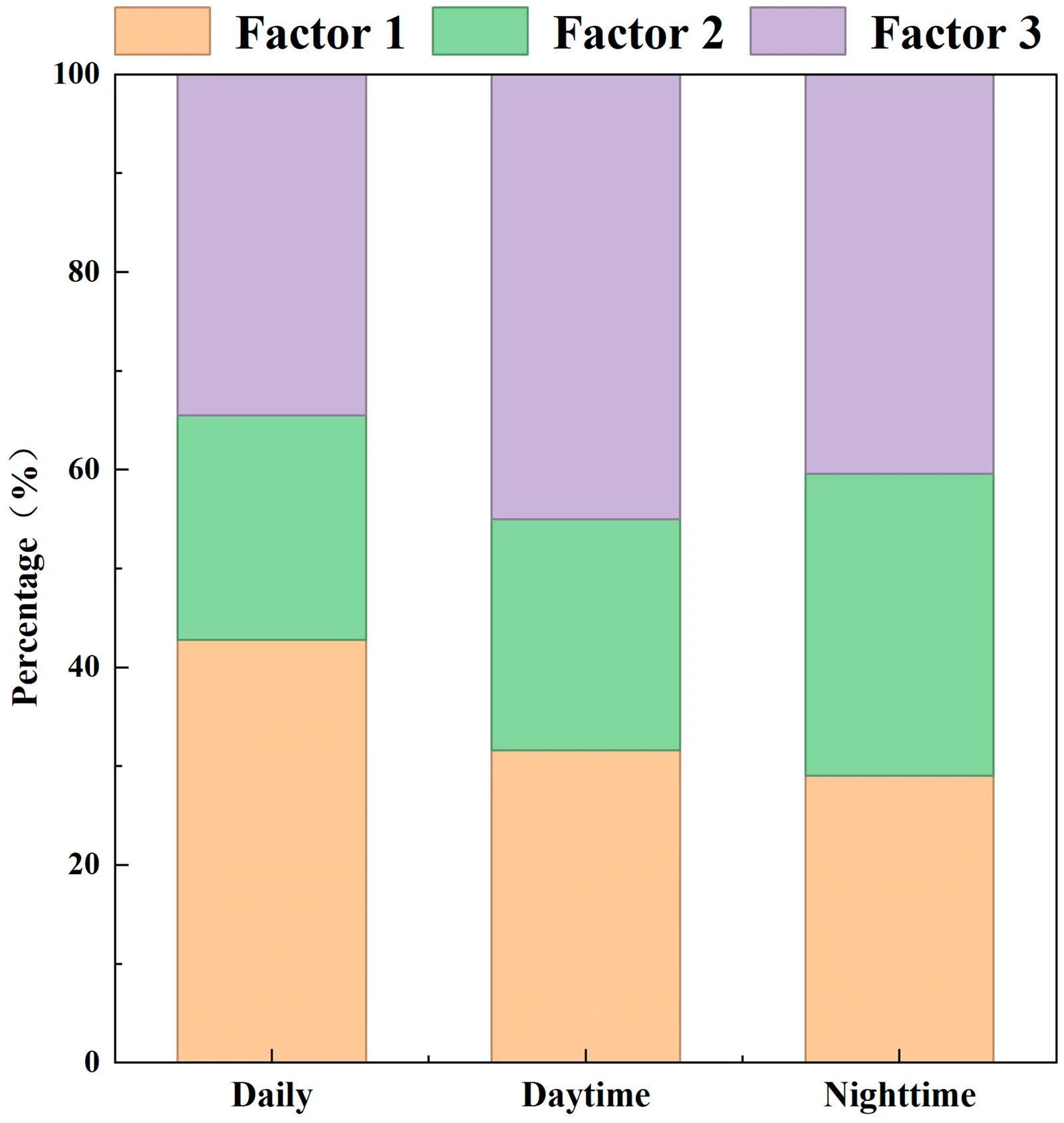
Relative source contributions to PM_2.5_-bound heavy metals for the daily run, daytime samples, and nighttime samples derived from the PMF model. Factor 1: mixed non-ferrous metal smelting and coal combustion; Factor 2: transportation; Factor 3: industrial combustion.

Source apportionment of the nine PM_2.5_-bound heavy metals was performed using PMF 5.0. Three independent PMF runs were conducted: a daily run on 40 daily-averaged samples (paired daytime and nighttime samples averaged by day), and daytime and nighttime runs on 40 samples each; contributions were normalized within each run, so daily proportions are not arithmetic averages of the daytime and nighttime values. The resulting factor profiles and contributions are presented in Figure 6a and Figure 7. Based on source characteristics and key indicator elements, three major sources were classified as a mixed source of non-ferrous metal smelting and coal combustion, a transportation source, and an industrial combustion source. To provide complementary evidence for the interpretation of the PMF-resolved factors, pairwise Pearson correlation coefficients among the nine heavy metals were calculated and displayed as a heatmap, while a network plot was constructed to visualize the relationships between individual metals and their PMF-derived factor contributions (Figure 6b).

Factor 1 accounted for 43% of the total PMF-resolved source contribution (Figure 7), with its loading spectrum characterized by marked enrichments of Ni (79%) and Pb (46%), while moderate contributions were also observed for Zn (36%), Cr (32%), V (27%), Cd (26%), Sb (23%), Co (22%), and Cu (21%) (Figure 6a). A significant positive correlation was observed between Pb and Ni (*p* < 0.01) (Figure 6b), indicating that these two heavy metals share identical or similar sources [61]. Since the ban on leaded gasoline in 2000, coal combustion and non-ferrous metal smelting have become important sources of Pb [62,63]; meanwhile, the loading combinations of Pb, V, Ni, and Cr point to high-temperature industrial activities such as coal combustion/smelting, building material manufacturing, and ferroalloy/electroplating processes, respectively [8,10,64]. The study area hosts highly polluting industries including smelting, coal-fired power generation, and building material manufacturing (Figure 1), whose emission characteristics are consistent with the above element combinations. Based on the loading spectral distribution and regional emission background, Factor 1 was identified as a mixed source of non-ferrous metal smelting and coal combustion.

Factor 2 was dominated by Cd (74%) and Sb (53%) with contributions significantly higher than those of other elements, while Zn (31%), Cu (26%), and Co (21%) also exhibited moderate loadings (Figure 6a). This factor accounted for 23% of the total PMF-resolved source contribution (Figure 7) and displayed a typical elemental spectral signature of traffic sources. It has been reported that Sb is widely regarded as a reliable tracer of brake wear, being added as antimony sulfide in friction materials [59]. Notably, no domestic coal-burning activities occur in winter in the study area; therefore, the high loading of Cd (74%) in this factor was mainly attributed to diesel/fuel oil combustion and high-temperature volatilization of lubricating oils from motor vehicles [23,65], and possibly also to the release of trace impurities from brake pads/tire materials [24]. Zn and Cu are also common tracer elements for non-exhaust traffic emissions, derived from zinc-containing additives during tire vulcanization and brake wear, respectively [20,60,66]. Moreover, a significant positive correlation was found between Cd and Sb (r = 0.73, *p* < 0.01) (Figure 6b), further supporting their common origin. The low contributions of Cr (17%) and Ni (6%) in this factor ruled out any significant influence from metallurgical or industrial combustion sources. Based on the above characteristics, Factor 2 was identified as the transportation source. Therefore, the low bulk EF of Sb does not necessarily conflict with its association with the transportation factor identified by PMF, because the two approaches characterize different aspects of elemental sources.

Factor 3 showed relatively high contributions from Co (56%), V (56%), Cu (53%), and Cr (52%), with moderate contributions from Zn (34%) and Sb (24%), whereas Ni (15%) and Pb (18%) contributed relatively less, and Cd (0%) was nearly absent (Figure 6a). This factor accounted for 34% of the total PMF-resolved source contribution and exhibited a characteristic high-temperature industrial profile. V and Ni are widely recognized as indicators of petroleum and residual-fuel combustion [25,67], while Co is also commonly associated with fuel-combustion processes [68]. The relatively high contributions of Cr and Cu further indicate the influence of high-temperature industrial and metallurgical processes, including industrial kilns and metal-processing activities [69]. Correlation analysis revealed strong positive correlations among Co, V, and Cr (r > 0.9, *p* < 0.01) (Figure 6b), further supporting their covariation within this factor.

To further explore the diurnal/nocturnal evolution characteristics of emission sources, the samples were divided into daytime and nighttime groups and separately input into the PMF model. Compared with the daily run, the characteristic element combinations of each factor remained unchanged in the daytime and nighttime samples: Factor 1 was dominated by Ni and Pb, Factor 2 by Cd and Sb, and Factor 3 by V, Co, and Cr (Figures S1 and S2). In terms of contribution proportions (Figure 7), Factor 1 was the largest contributor in the daily run (43%), whereas Factor 3 was the largest contributor in both the daytime (45%) and nighttime (40%) runs. Each apportionment reflects the source structure of its own dataset [34,35]. The daytime and nighttime runs are therefore used to examine how the relative importance of the three sources changed between the two periods. The contribution of Factor 2 (transportation source) was nominally higher at night (31%) than during the daytime (23%); the contribution of Factor 1 (mixed non-ferrous metal smelting and coal combustion source) was comparable between day and night (32% during daytime vs. 29% at night). It is noteworthy that, although all volumetric elemental concentrations were higher at night than during the daytime, the changing trends in relative contribution proportions of the factors were not entirely consistent: the contribution of Factor 3 was nominally higher in the daytime, and although its contribution proportion decreased somewhat at night, the concentrations of characteristic elements such as V, Co, and Cr were still higher at night, reflecting the overall effect of pollutant accumulation caused by a lowered nocturnal boundary layer; the contribution of Factor 2 was higher at night, consistent with the higher nighttime concentrations of traffic-related elements such as Cd and Sb; the contribution of Factor 1 remained roughly unchanged between day and night, indicating that mixed non-ferrous metal smelting and coal combustion source are relatively continuous over time and are less influenced by daytime/nocturnal differences.

Although the source categories identified here, including industrial combustion, traffic emissions, and smelting/combustion-related sources, have also been reported in other urban environments, their relative contributions are strongly dependent on local industrial structure and meteorological conditions. Multi-season studies have shown that the dominant sources of individual PM_2.5_-bound heavy metals may change substantially throughout the year. For example, year-long measurements in Beijing demonstrated obvious seasonal variability in the major sources of K, Fe, Zn, Pb, Mn, Cu, and Ni, whereas the dominant sources of several other elements remained relatively stable [70]. Therefore, the source proportions resolved in the present study should be interpreted specifically for wintertime Laibin. Nevertheless, the paired daytime–nighttime observations provide additional information on short-term source dynamics, particularly the enhanced nighttime contribution of traffic emissions and the persistent influence of industrial-related sources.

### 3.5. Health Risk Assessment

**Figure 8:**
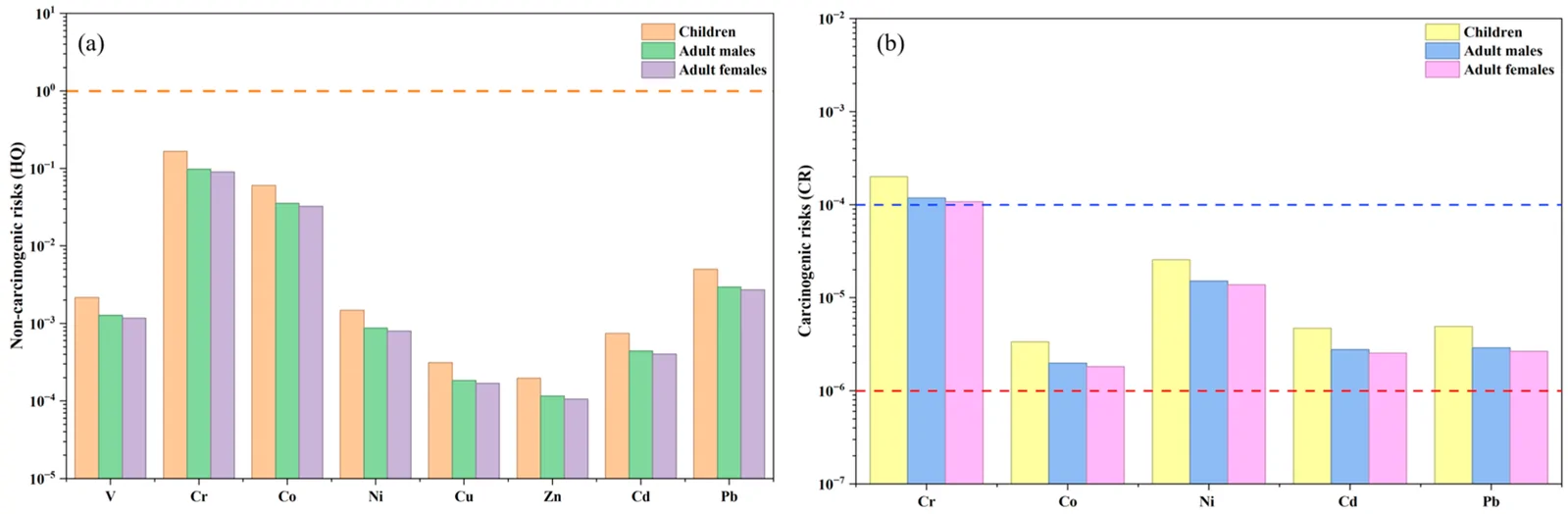
Non-cancer (**a**) and cancer risks (**b**) for PM_2.5_-bound heavy metals (lines represent the threshold values for non-cancer and cancer risks). The orange dotted line in panel (**a**) represents the non-carcinogenic risk threshold of *HQ* = 1. The blue dotted line and red dotted line in panel (**b**) represent carcinogenic risk thresholds of 1 × 10 ^−4^ and 1 × 10 ^−6^, respectively.

The non-carcinogenic hazard index (HI) for the eight heavy metals exhibited a consistent ranking across demographic groups: children exhibited the highest values, followed by adult males and then adult females, a pattern that corroborates earlier observations [71]. All population groups exhibited *HI* values below the acceptable threshold of 1, specifically 2.36 × 10^−1^ for children, 1.39 × 10^−1^ for adult males, and 1.28 × 10^−1^ for adult females, suggesting that the non-carcinogenic risks from inhalation exposure remained within tolerable levels. Among the individual elements, the highest hazard quotient (HQ) was consistently observed for Cr in all three populations (children: 1.66 × 10^−1^; adult males: 9.8 × 10^−2^; adult females: 8.9 × 10^−2^) (Figure 8a), suggesting that long-term exposure to Cr may pose potential non-carcinogenic health effects on susceptible populations, particularly children.

With respect to carcinogenic risk, the average *CR* values for all populations were slightly above the threshold of 1 × 10^−4^ (Table S4), indicating that the risk has exceeded the generally acceptable level and should be regarded as a significant concern that requires priority emission reduction measures. Notably, the highest contribution to carcinogenic risk was also attributed to Cr across all three populations, with values all exceeding 1 × 10^−4^, demonstrating that Cr was the predominant carcinogenic risk factor in the study area (Figure 8b). As documented in the literature, hexavalent chromium (Cr (VI)) has been significantly associated with an elevated risk of lung cancer following inhalation exposure [72]. Given that Cr was found to dominate both non-carcinogenic and carcinogenic risks, and that the highest exposure risk was observed in children, priority targeted control measures should be implemented by local governments, with particular emphasis placed on the reduction of chromium emissions from major anthropogenic sources, such as coal combustion [68] and industrial combustion [73].

A key limitation of the present health-risk assessment is the temporal representativeness of the exposure data. Because the concentrations used in the exposure calculations were obtained exclusively during the winter campaign, the resulting *HQ*, *HI*, and *CR* values do not represent measured annual-average exposure concentrations. Previous multi-season studies have shown that PM_2.5_-bound heavy metal concentrations and source-specific health risks can vary substantially with season owing to changes in emission intensity, meteorological dispersion, and source composition. Chen et al. (2021), for example, reported seasonal differences in source-specific health risks and indicated that estimates based on shortened sampling periods may deviate from those derived from longer datasets [74]. Yang et al. (2022) similarly observed seasonal changes in the dominant sources of several PM_2.5_-bound heavy metals based on year-long measurements [70]. Therefore, the *HQ*, *HI*, and *CR* values reported here should be interpreted as screening-level estimates derived from the wintertime concentration scenario and the exposure assumptions adopted in the HRA model, rather than as direct estimates of annual exposure. Future multi-season observations using a consistent sampling and analytical protocol are needed to better constrain estimates of annual-average exposure and to characterize seasonal variability in source-specific health risks.

## 4. Conclusions

Based on a winter campaign lasting 42 days in 2025, three main findings emerge. (1) The nine target heavy metals averaged 363.0 ng/m^3^, dominated by Zn, Cr, and Ni, and showed nocturnal volumetric accumulation. When normalized to PM_2.5_ mass (mean of ratios), all nine metals were nominally higher in daytime on a mass-normalized basis, though none reached statistical significance; the mean Cu/PM_2.5_ ratio was nearly identical between day and night, consistent with the nocturnal rise in volumetric Cu being driven mainly by PM_2.5_ mass accumulation rather than disproportionate brake-wear particle input. EF results indicate strong anthropogenic influences on Cd and Ni, while other metals reflect mixed contributions from anthropogenic, natural, and crustal sources. (2) PMF analysis preliminarily resolved three major sources: mixed non-ferrous smelting and coal combustion (43%), traffic (23%), and industrial combustion (34%) in the daily run. In the separate daytime and nighttime runs, the industrial combustion source was the largest in both periods, traffic contributions increased at night, and the mixed non-ferrous metal smelting and coal combustion source remained relatively stable between day and night. Industrial combustion showed the highest Cr loading and was therefore identified as the largest source contributor to Cr, followed by the mixed non-ferrous metal smelting and coal combustion source. (3) The noncancer hazard index was below 1 for all groups (children > adult males > adult females), whereas total carcinogenic risk slightly exceeded 1 × 10^−4^ across all populations, with Cr dominating both noncancer and carcinogenic risks. These risk estimates represent conservative screening values, as they rely on potential inhaled doses and do not account for exhaled or undeposited particles or metal bioaccessibility after deposition. Since Cr is the primary driver of both noncancer and carcinogenic risks and stems largely from industrial combustion and mixed non-ferrous smelting and coal combustion sources, one precautionary interim measure is to prioritize Cr emission controls for these industrial facilities during winter pollution episodes, especially under stagnant meteorological conditions, while sustaining existing PM_2.5_ control strategies. However, since the source apportionment and risk estimates are derived solely from this winter campaign lasting 42 days in 2025, they should not be generalized to annual conditions or conditions in other seasons. Monitoring across multiple seasons is required to verify these source patterns and evaluate whether such control measures should be extended to other periods.

## Figures and Tables

**Table 1:** Exposure parameters used in the health risk assessment of PM_2.5_-bound heavy metals for children, adult men, and women.

Parameter	Unit	Children	Adult Males	Adult Females
*InhR*	m^3^/day	7.5	17.3	14
*EF*	Days	350	350	350
*ED* (carcinogenic)	Years	6	72	79
*ED* (non-carcinogenic)	Years		24	24
*BW*	Kg	15.9	62.2	55
*AT* (carcinogenic)	D	6 × 350	72 × 350	79 × 350
*AT* (non-carcinogenic)	D		24 × 350	24 × 350

**Table 2:** Reference doses (RfD) and slope factors (SF) for non-carcinogenic and carcinogenic metals via inhalation exposure.

Element	*RfD* Inhale	*SF* Inhale
V	7.00 × 10^−3^	
Cr	2.86 × 10^−5^	41
Co	5.71 × 10^−6^	9.8
Ni	2.06 × 10^−2^	8.4 × 10^−1^
Cu	4.02 × 10^−2^	
Zn	3.00 × 10^−1^	
Cd	1.00 × 10^−3^	6.3
Pb	3.52 × 10^−3^	4.2 × 10^−2^

## Data Availability

The raw data supporting the findings of this study are provided in Table S5 of the Supplementary Materials.

## Supplementary Materials

The following supporting information can be downloaded at https://www.mdpi.com/article/10.3390/toxics14100923/s1. Table S1: Descriptive statistics of PM_2.5_, heavy metals, and meteorological factors in the study area during winter; Table S2: PM_2.5_ and heavy metal concentrations in other cities of southern China in winter; Table S3: Day–night comparisons of PM2.5-bound heavy metals (Wilcoxon signed-rank test, *n* = 40 paired samples); Table S4: Non-carcinogenic and carcinogenic risks via inhalation exposure to PM_2.5_-bound heavy metals for children, adult males, and adult females; Table S5: Raw data for PM_2.5_-bound metal elements in Laibin, Guangxi, China, during winter 2025; Figure S1: Source profiles and percentage contributions of three sources to PM_2.5_-bound heavy metals resolved by the PMF model during daytime; Figure S2: Source profiles and percentage contributions of three sources to PM_2.5_-bound heavy metals resolved by the PMF model during nighttime.

## Abbreviations

The following abbreviations are used in this manuscript:

PM_2.5_	Fine particulate matter with an aerodynamic diameter ≤ 2.5 μm
IARC	International Agency for Research on Cancer
ROS	Reactive Oxygen Species
PMF	Positive Matrix Factorization
EF	Enrichment Factor
ICP-MS	Inductively Coupled Plasma Mass Spectrometry
HRA	Health Risk Assessment
U.S. EPA	United States Environmental Protection Agency
HQ	Hazard Quotient
CR	Carcinogenic Risk
ADD	Average Daily Dose
RfD	Reference Dose
SF	Slope Factor
HI	Hazard Index
LADD	Lifetime Average Daily Dose
MDL	Method Detection Limit
WHO	World Health Organization
EU	European Union
