# Supplementary material for: Source Apportionment and Health Risk Assessment of PM2.5-Bound Heavy Metals in Wintertime Laibin, Guangxi, China
Source: Toxics. 2026 Oct 19;14(10):923. doi: 10.3390/toxics14100923 (PMC13709916; doi:10.3390/toxics14100923)
Supplement: Supplementary file 1 [file toxics-14-00923-s001.zip › toxics-4576414-supplementary.pdf]

Supplementary File

Source Apportionment and Health Risk Assessment of $PM_{2.5}$-Bound Heavy Metals in Wintertime Laibin, Guangxi, China

Rui Qu [1], Jingying Mao [2], Jian Gao [1,*], Kuiquan Duan [1], Yuanguan Gao [1], Haisheng Li [1,*], Chuan Nong [2], Jiahui Lu [2] and Hexiao Chen [2]

1 State Key Laboratory of Environmental Criteria and Risk Assessment, Chinese Research Academy of Environmental Sciences, Beijing 100012, China; qurui@craes.org.cn (R.Q.); duankuiquan@craes.org.cn (K.D.); gaoyg@craes.org.cn (Y.G.)
2 Guangxi Research Academy of Environmental Sciences, Nanning 530022, China; 13978185061@163.com (J.M.); nongchuan@gxhky.cn (C.N.); lujiahui@gxhky.cn (J.L.); chenhexiao@gxhky.cn (H.C.)
* Correspondence: gaojian@craes.org.cn (J.G.); lihs@craes.org.cn (H.L.)

Table S1 Descriptive statistics of $PM_{2.5}$, heavy metals, and meteorological factors in the study area during winter.

	Unit	Median	Mean	SD	CV(unitless)	BV[1]	Min	Max
$PM_{2.5}$	μg/m³	40.9	47.6	33.3	0.699	-	6.8	142.5
THMs	ng/m³	323.2	363.0	145.7	0.401		175.6	857.9
Co	ng/m³	0.6	0.7	0.5	0.639	4.8	0.4	3.2
Cd	ng/m³	0.9	1.6	2.4	1.506	0.0791	0.2	15.1
Sb	ng/m³	2.9	3.7	2.5	0.685	2120	1.2	11.4
Cu	ng/m³	12.2	27.8	43.3	1.559	21.4	5.6	187.0
V	ng/m³	27.7	32.1	16.3	0.508	106.9	20.4	120.4
Pb	ng/m³	31.2	37.2	20.0	0.538	20.5	10.0	85.9
Ni	ng/m³	69.7	64.5	45.0	0.698	17.4	10.3	174.0
Cr	ng/m³	57.5	70.6	42.2	0.598	64.3	47.7	306.5
Zn	ng/m³	104.9	124.9	55.4	0.444	50.8	56.0	307.8
TP	℃	12.3	13.0	2.9	0.224	-	9.0	19.7
RH	%	71.1	69.6	17.4	0.250	-	38.9	94.1
AP	hPa	1003.2	1003.3	4.4	0.004	-	995.7	1012.2
RF	mm/h	0.0	0.1	0.1	1.652	-	0.00	0.5
WS	m/s	3.0	3.0	1.3	0.434	-	1.0	5.9

Abbreviations: BV, background value; CV, coefficient of variation; SD, standard deviation; THMs, total heavy metals; TP, temperature; RH, relative humidity; AP, atmospheric pressure; RF, rainfall; WS, wind speed. CV values are dimensionless coefficients of variation, not percentages, and are reported to three decimal places to avoid rounding small but non-zero variability (e.g., AP: CV = 0.004) to zero.

Table S2. $PM_{2.5}$ and heavy metal concentrations in other cities of southern China in winter.

City	$PM_{2.5}$	Co	Cd	Sb	Cu	V	Pb	Ni	Cr	Zn
	μg/m³	ng/m³	ng/m³	ng/m³	ng/m³	ng/m³	ng/m³	ng/m³	ng/m³	ng/m³
Chongqing[2]	97.1	-	-	-	15.8	0.6	37.9	1.4	4.3	94.2
Huangshi[3]	71.1	1.8	11.2	-	52.2	1.3	51.4	3.5	10.8	150.0
Fuzhou[4]	59.8	-	-	-	164.9	3.7	46.6	3.4	10.1	232.6
Suzhou[5]	57.0	-	0.7	4.2	-	-	21.9	3.2	4.6	-
Nanning[6]	65.0	-	-	-	-	-	-	-	-	-

Table S3. Day–night comparisons of $PM_{2.5}$-bound heavy metals (paired Wilcoxon signed-rank test, n = 40 paired samples).

	Daytime mean ± SD	Nighttime mean ± SD	p (volumetric)	Daytime norm. mean ± SD	Nighttime norm. mean ± SD	p (normalized)
$PM_{2.5}$	45.3±36.3	49.8±35.4	0.154			
V	27.3±9.7	37.0±31.7	0.002 **	1.5±2.5	1.3±1.2	0.610
Cr	58.9±21.8	82.2±82.7	0.005 **	3.2±5.7	2.8±2.7	0.657
Co	0.6±0.3	0.8±0.9	0.011 *	0.03±0.06	0.026±0.024	0.830
Ni	56.1±44.7	72.9±58.7	<0.001 ***	3.7±8.2	3.0±3.5	0.436
Cu	21.7±44.6	33.9±59.3	0.007 **	0.7±1.3	0.7±0.8	0.211
Zn	112.3±68.9	137.4±79.0	0.012 *	6.3±14.6	4.5±4.1	0.707
Cd	1.4±2.8	1.8±2.2	0.016 *	0.04±0.07	0.037±0.029	0.282
Sb	3.2±2.4	4.1±3.4	0.019 *	0.14±0.24	0.108±0.077	0.468
Pb	32.7±24.1	41.8±31.8	0.116	1.6±4.1	1.3±1.2	0.778
THMs	314.3±160.8	411.8±242.8	0.001 **	17.2±36.4	13.9±12.4	0.582

Note: p-values from paired Wilcoxon signed-rank test (Shapiro–Wilk tests indicated non-normality for all variables). * $p < 0.05$, ** $p < 0.01$, *** $p < 0.001$. $PM_{2.5}$ concentrations are in μg/m³; heavy metal concentrations are in ng/m³; normalized concentrations are in ng/μg $PM_{2.5}$. Norm. = $PM_{2.5}$-normalized (mean of individual metal/$PM_{2.5}$ ratios). THMs = sum of the nine measured metals.

Table S4. Non-carcinogenic and carcinogenic risks via inhalation exposure to PM2.5-bound heavy metals for children, adult males, and adult females.

Element	Children		Adult female		Adult male	
	Hq_{inh}	Cr_{inh}	Hq_{inh}	Cr_{inh}	Hq_{inh}	Cr_{inh}
Co	6.02×10^{-2}	3.37×10^{-6}	3.25×10^{-2}	1.82×10^{-6}	3.55×10^{-2}	1.99×10^{-6}
Cd	7.46×10^{-4}	4.70×10^{-6}	4.03×10^{-4}	2.54×10^{-6}	4.40×10^{-4}	2.77×10^{-6}
Cu	3.26×10^{-4}	-	1.76×10^{-4}	-	1.92×10^{-4}	-
V	2.16×10^{-3}	-	1.17×10^{-3}	-	1.28×10^{-3}	-
Pb	4.99×10^{-3}	7.37×10^{-7}	2.69×10^{-3}	3.98×10^{-7}	2.94×10^{-3}	4.35×10^{-7}
Ni	1.48×10^{-3}	2.56×10^{-5}	7.97×10^{-4}	1.38×10^{-5}	8.71×10^{-4}	1.51×10^{-5}
Cr	1.66×10^{-1}	1.95×10^{-4}	8.97×10^{-2}	1.05×10^{-4}	9.80×10^{-2}	1.15×10^{-4}
Zn	1.96×10^{-4}	-	1.06×10^{-4}	-	1.16×10^{-4}	-
Total	2.36×10^{-1}	2.29×10^{-4}	1.28×10^{-1}	1.24×10^{-4}	1.39×10^{-1}	1.35×10^{-4}

Table S5. Raw data for $PM_{2.5}$-bound metal elements in Laibin, Guangxi, China, during winter 2025.

	Heavy metal concentration										Parameters of environmental factors			Air quality parameters
Unit	ng/m³										℃	%	m/s	μg/m³
Sampling code	V	Cr	Mn	Co	Ni	Cu	Zn	Cd	Sb	Pb	Temperature	Relative Humidity	Wind Speed	$PM_{2.5}$
XBQ-0117-day	34.282	72.825	67.402	0.775	13.364	13.209	148.362	1.627	4.687	29.208	15.65	37.28	1.84	68.92
XBQ-0117-night	38.190	79.622	74.218	0.721	12.430	12.754	118.892	1.693	3.927	29.111	11.94	49.36	1.94	75.75
XBQ-0118-day	37.687	83.162	55.171	0.690	12.779	10.304	105.880	2.028	4.868	35.942	16.75	40.55	1.21	81.42
XBQ-0118-night	27.344	57.705	105.982	0.641	12.710	23.082	102.210	2.414	5.205	47.145	12.96	50.30	2.05	79.67
XBQ-0119-day	27.250	57.055	87.712	0.639	14.775	13.072	104.317	2.257	5.578	37.043	18.78	37.60	1.60	74.92
XBQ-0119-night	26.127	50.272	59.813	0.587	15.192	77.060	118.892	2.569	4.991	35.594	12.90	60.05	1.32	98.92
XBQ-0120-day	29.624	56.887	74.418	0.644	11.807	10.733	154.560	2.791	7.692	46.153	19.55	41.50	1.12	88.17
XBQ-0120-night	30.115	55.466	101.625	0.633	13.550	11.391	134.384	2.978	6.738	39.459	12.85	65.98	0.93	87.75
XBQ-0121-day	28.271	60.755	197.655	0.729	14.095	17.133	259.220	18.064	11.381	97.613	19.79	46.51	1.95	124.33
XBQ-0121-night	39.919	86.698	69.858	1.060	21.020	20.334	230.156	12.100	11.477	62.997	15.94	62.46	1.84	160.64
XBQ-0122-day	26.370	55.752	44.358	0.855	13.511	11.679	96.447	3.581	4.476	33.126	20.65	51.69	2.52	125.30
XBQ-0122-night	74.232	159.279	53.093	1.475	30.676	21.041	175.011	3.540	5.703	35.690	18.69	65.42	0.71	72.89
XBQ-0124-day	27.803	55.461	82.461	0.547	13.865	11.129	82.461	0.912	2.737	16.711	19.22	84.65	1.96	71.25
XBQ-0124-night	24.291	46.576	32.281	0.573	11.751	10.892	91.718	2.114	3.260	15.907	17.83	85.28	3.61	39.09
XBQ-0126-day	24.264	51.663	23.943	0.570	11.865	6.770	75.890	0.178	1.390	7.945	10.44	62.92	7.29	7.25
XBQ-0126-night	29.216	54.869	194.180	0.499	10.297	8.302	65.914	0.285	2.316	15.606	8.93	55.51	4.55	21.33
XBQ-0127-day	23.227	46.025	130.584	0.535	10.811	8.170	73.498	0.464	1.677	10.561	11.77	39.48	3.81	22.09
XBQ-0127-night	26.189	56.093	52.163	0.536	11.433	52.163	84.318	0.965	2.751	28.047	11.86	39.17	2.38	47.08
XBQ-0128-day	24.117	50.019	88.963	0.607	13.362	24.581	104.683	0.643	1.858	17.578	15.12	35.54	1.69	37.25

XBQ-0128-night	33.284	64.490	81.329	1.111	19.096	349.318	165.523	1.648	3.439	37.261	13.39	53.50	1.30	83.42
XBQ-0129-day	34.740	62.969	96.600	1.109	12.952	230.767	118.424	1.324	3.649	45.080	16.63	41.20	1.86	137.17
XBQ-0129-night	25.474	52.520	39.301	0.750	12.612	95.037	85.748	1.036	2.465	22.437	14.60	50.34	1.96	97.00
XBQ-0130-day	30.405	61.452	92.179	0.857	14.970	190.074	110.757	1.250	3.323	37.157	16.25	64.10	2.90	70.45
XBQ-0130-night	31.699	61.180	86.940	0.966	19.177	150.624	221.822	1.825	5.975	31.699	16.36	84.58	3.39	95.82
XBQ-0131-day	24.688	48.233	58.237	0.643	11.290	25.224	79.674	0.500	1.822	11.254	18.42	78.85	4.78	45.45
XBQ-0131-night	25.493	52.910	54.513	0.641	14.964	68.943	95.665	1.390	5.558	19.026	18.48	83.94	1.13	40.25
XBQ-0201-day	20.801	43.528	230.484	0.535	10.347	11.524	69.217	0.571	1.570	41.387	15.84	89.20	3.14	28.33
XBQ-0201-night	24.406	52.731	54.869	0.570	10.154	7.304	42.755	0.178	1.568	12.007	12.61	83.31	4.25	13.27
XBQ-0202-day	25.796	54.869	36.342	0.428	63.776	9.050	100.831	0.178	1.425	8.373	11.06	77.56	5.29	7.45
XBQ-0202-night	27.755	58.432	16.924	0.392	81.947	14.786	112.232	0.178	1.140	11.544	9.34	80.09	5.40	6.17
XBQ-0203-day	22.945	48.812	41.330	0.428	60.926	15.534	86.579	0.214	1.033	12.755	10.26	72.90	6.33	7.45
XBQ-0203-night	24.121	52.019	26.686	0.428	67.696	23.159	105.819	0.499	1.817	25.261	9.95	67.05	4.39	23.58
XBQ-0204-day	27.366	58.870	160.554	0.428	65.649	20.979	97.403	0.892	2.819	43.171	13.99	47.85	2.74	43.58
XBQ-0204-night	28.336	64.042	158.496	0.680	114.489	40.429	198.567	4.150	5.367	67.620	11.49	66.08	1.08	74.42
XBQ-0205-day	24.264	53.800	37.411	0.392	65.558	14.715	87.648	1.105	1.888	21.556	13.45	55.38	1.85	84.82
XBQ-0205-night	26.116	57.720	40.974	0.428	67.339	7.660	87.292	0.392	1.390	12.648	11.37	77.79	1.54	84.80
XBQ-0206-day	25.332	49.881	96.199	0.570	55.938	7.233	95.487	0.321	1.140	19.418	10.29	92.73	2.97	15.64
XBQ-0206-night	29.897	66.918	29.466	0.648	67.278	15.650	87.785	0.324	1.331	23.565	9.15	91.83	4.94	6.92
XBQ-0207-day	25.226	52.375	122.565	0.463	62.708	17.850	79.453	0.285	1.425	17.565	9.45	79.30	5.31	9.18
XBQ-0207-night	23.184	51.520	137.387	0.501	65.831	13.417	102.682	0.859	3.649	44.007	9.03	63.87	4.72	21.33
XBQ-0208-day	25.083	55.225	64.133	0.641	68.408	8.551	102.256	0.570	1.211	30.570	10.01	39.91	5.57	31.00
XBQ-0208-night	29.147	61.473	102.036	0.627	78.200	15.807	126.709	1.255	1.840	106.218	8.07	42.97	3.87	29.92
XBQ-0209-day	24.743	54.136	77.026	0.436	65.399	10.646	128.618	1.780	2.035	63.583	11.29	34.65	2.39	19.82

XBQ-0209-night	25.286	55.018	63.398	0.437	64.855	9.765	148.657	1.640	2.696	47.731	12.32	43.07	1.48	44.75
XBQ-0210-day	21.271	46.674	45.249	0.356	61.995	9.941	107.957	1.603	7.447	36.698	13.64	50.59	1.16	49.50
XBQ-0210-night	44.609	101.977	91.827	0.870	95.211	15.659	193.804	2.900	9.618	48.330	11.09	67.77	1.08	81.55
XBQ-0211-day	27.133	60.874	36.376	0.445	77.948	9.502	117.293	0.668	2.821	10.467	15.88	64.43	4.55	101.91
XBQ-0211-night	70.014	153.805	264.205	1.321	183.056	34.818	498.215	7.643	16.607	119.836	18.37	76.75	2.99	43.18
XBQ-0212-day	30.767	66.326	35.516	0.599	76.167	11.468	108.260	0.556	2.696	32.478	18.19	82.27	2.94	68.64
XBQ-0212-night	24.652	54.307	51.449	0.393	61.810	10.683	88.606	0.857	3.573	27.296	12.48	72.12	6.03	24.17
XBQ-0213-day	25.118	52.448	94.192	0.500	64.578	9.062	94.192	0.963	2.819	31.683	14.50	56.04	4.03	39.45
XBQ-0213-night	215.747	560.589	138.526	5.954	278.232	28.531	216.337	2.594	6.189	120.253	12.98	61.14	2.25	60.83
XBQ-0214-day	25.176	57.285	47.433	0.511	77.718	8.903	90.853	1.058	2.335	32.036	15.32	62.84	1.68	66.18
XBQ-0214-night	48.872	104.471	301.964	0.787	140.249	15.313	150.982	0.572	3.721	30.053	13.23	77.23	2.58	101.27
XBQ-0215-day	20.925	47.624	21.286	0.361	72.157	7.216	68.549	0.361	1.118	24.317	11.45	78.77	4.16	12.56
XBQ-0215-night	48.502	105.988	114.411	0.702	141.785	12.564	153.016	0.702	3.510	43.308	11.36	85.09	3.49	17.36
XBQ-0216-day	23.784	53.423	36.957	0.439	69.889	5.891	68.425	0.183	1.281	10.428	12.45	80.12	3.62	20.09
XBQ-0216-night	66.406	152.026	70.840	1.267	199.534	21.326	262.879	2.006	5.279	156.249	12.00	88.41	2.77	27.10
XBQ-0217-day	28.464	65.339	32.294	0.488	74.351	6.872	97.633	0.338	2.291	28.238	10.78	82.40	3.95	22.50
XBQ-0217-night	32.406	72.128	52.550	0.515	98.918	10.510	110.768	0.721	3.194	34.415	10.01	87.25	3.96	22.67
XBQ-0218-day	24.492	55.071	39.491	0.399	73.548	6.956	95.287	0.942	2.283	30.832	10.51	82.23	3.50	13.18
XBQ-0218-night	23.838	53.590	73.547	0.554	72.808	8.427	99.418	0.554	1.737	32.819	10.35	87.58	2.28	55.83
XBQ-0219-day	20.337	46.025	42.101	0.321	67.076	5.744	75.282	0.535	1.320	32.004	12.00	88.11	2.28	22.64
XBQ-0219-night	25.637	58.751	23.459	0.370	76.417	5.505	79.293	0.329	1.150	21.159	11.24	93.11	2.46	25.42
XBQ-0220-day	22.795	50.734	36.443	0.357	70.742	7.038	75.744	0.393	1.858	23.974	13.03	85.92	3.42	22.73
XBQ-0220-night	26.491	58.441	66.515	0.423	75.358	5.652	77.280	0.423	1.769	23.030	10.83	92.53	4.33	8.58
XBQ-0221-day	22.518	50.594	30.463	0.428	67.696	7.233	74.465	0.356	9.157	19.988	11.21	85.20	3.60	7.73

XBQ-0221-night	24.434	54.172	51.141	0.606	73.113	7.084	98.115	0.379	1.364	33.261	10.71	85.26	2.72	17.83
XBQ-0222-day	26.545	58.513	105.966	0.392	69.930	6.315	86.342	0.214	2.854	14.628	10.44	94.22	3.20	15.56
XBQ-0222-night	33.058	73.945	51.713	0.532	97.627	8.168	116.476	0.435	1.692	27.548	9.73	94.02	4.31	7.64
XBQ-0223-day	81.613	182.968	75.354	1.806	262.415	41.409	480.292	2.407	5.417	133.615	9.69	84.39	5.70	5.09
XBQ-0223-night	26.458	59.898	21.424	0.661	85.622	9.407	103.628	0.661	1.727	38.217	9.92	73.35	4.77	14.42
XBQ-0224-day	19.356	44.972	35.834	0.540	79.151	8.167	106.134	0.756	2.051	44.612	10.67	68.28	4.03	29.18
XBQ-0224-night	21.384	50.364	40.993	0.702	92.472	82.193	97.426	1.321	2.725	60.685	10.77	72.72	2.37	81.75
XBQ-0225-day	23.480	55.225	88.361	1.033	101.900	10.796	108.669	0.998	2.743	65.202	10.27	82.35	2.72	71.36
XBQ-0225-night	22.173	52.665	30.111	1.221	104.567	7.136	102.658	0.382	2.023	23.279	10.31	83.50	2.56	27.33
XBQ-0226-day	30.819	75.890	34.632	0.998	94.061	7.518	95.843	0.499	2.245	17.708	10.30	88.09	2.02	12.89
XBQ-0226-night	30.936	70.967	54.500	1.215	112.921	8.900	92.140	1.059	1.490	32.621	11.22	94.25	0.56	23.55
XBQ-0227-day	22.090	53.088	70.546	1.211	94.418	10.226	179.215	1.995	7.090	33.812	12.81	94.64	1.60	30.27
XBQ-0227-night	22.825	52.770	46.792	0.598	103.801	14.347	252.165	2.663	12.771	27.499	16.80	91.27	3.04	47.70

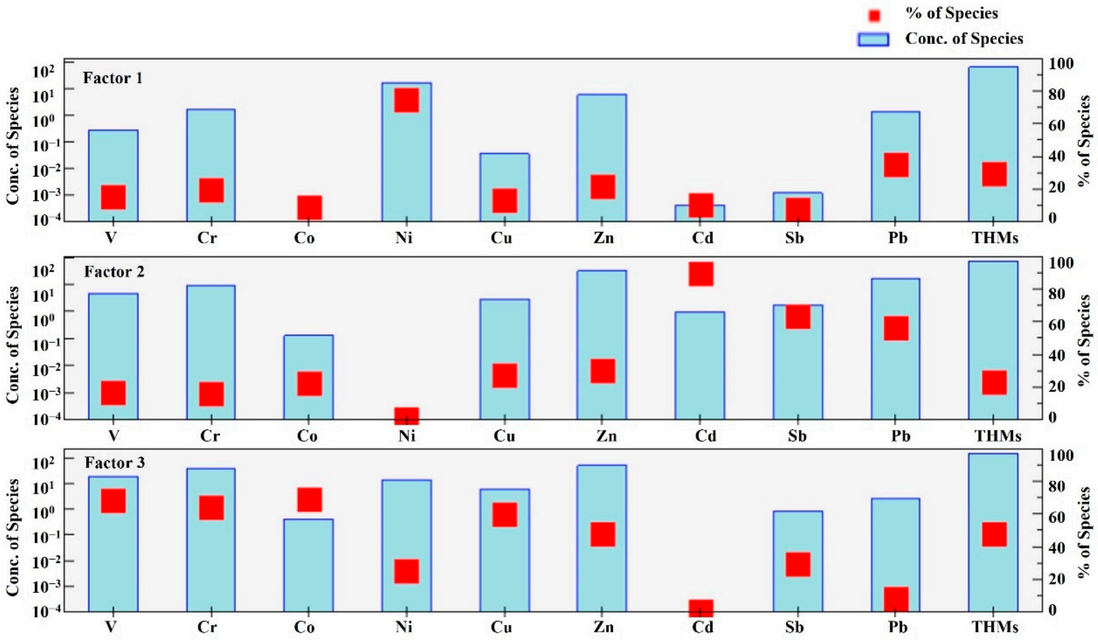

Figure S1. Source profiles and percentage contributions of three sources to $PM_{2.5}$-bound heavy metals resolved by the PMF model during daytime.

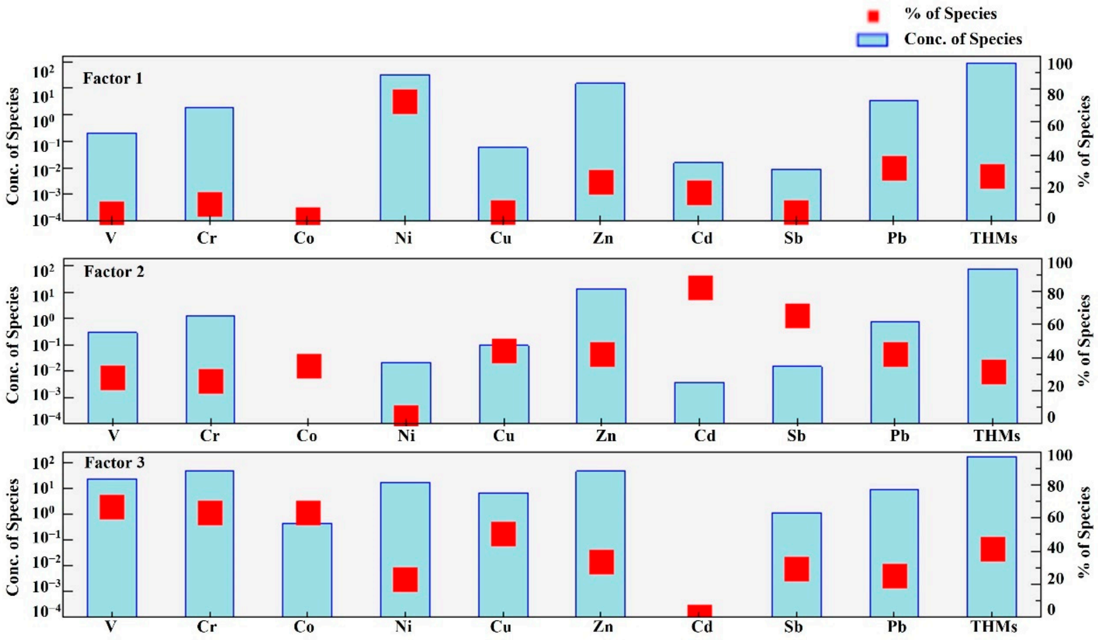

Figure S2. Source profiles and percentage contributions of three sources to $PM_{2.5}$-bound heavy metals resolved by the PMF model during nighttime.

The residual for heavy metal n in sample m, represented as E_{mn}, is obtained through the minimization of the objective function Q:

$$Qn\sum_{m=1}^{i}\sum_{b=1}^{j}\left(\frac{X_{mn}-\sum_{\alpha=1}^{\beta}G_mF_{n\alpha}}{U_{mn}}\right) \tag{S1}$$

The uncertainty (U_{mn}) is calculated as follows:

For samples with concentrations lower than the detection limits:

$$X_{mn}=\frac{MDL}{2}\,,U_{mn}=\frac{5}{6}MDL \tag{S2}$$

For concentrations above the detection limits[7]

$$X_{mn}=V_{mn} \tag{S3}$$

$$\text{if } X_{mn}\leq 3MDL, U_{mn}=\frac{1}{3}MDL+0.2*V_{mn} \tag{S4}$$

$$\text{if } X_{mn}>3MDL, U_{mn}=\frac{1}{3}MDL+0.1*V_{mn} \tag{S5}$$

For missing values:

$$X_{mn}=\tilde{V}_{mn}\,,U_{mn}=4\tilde{V}_{mn} \tag{S6}$$

where V_{mn} is the measured concentration, U_{mn} is the measurement precision, MDL is the minimum detection limit, and $\tilde{V}_{mn}$ is the geometric mean of the observed value V_{mn}.

References

1. Station, C.E.M. Background values of elements in soils of China. *Chin. Environ. Sci. Press Beijing China* **1990**, 418–451
2. Han, Y.; Wang, Z.; Zhou, J.; Che, H.; Tian, M.; Wang, H.; Shi, G.; Yang, F.; Zhang, S.; Chen, Y. PM2.5-Bound Heavy Metals in Southwestern China: Characterization, Sources, and Health Risks. *Atmosphere* **2021**, *12*, 929.
3. Liu, Z.; Zhan, C.; Liu, H.; Liu, S.; Quan, J.; Liu, X.; Zhang, J.; Qu, C. Source-Specific Health Risk of PM2.5-Bound Metals in a Typical Industrial City, Central China, 2021–2022. *Atmosphere* **2023**, *14*, 1406.
4. Xu, L.L.; Chen, X.Q.; Chen, J.S.; Zhang, F.W.; He, C.; Zhao, J.P.; Yin, L.Q. Seasonal variations and chemical compositions of PM2.5 aerosol in the urban area of Fuzhou, China. *Atmos. Res.* **2012**, *104*, 264–272. https://doi.org/10.1016/j.atmosres.2011.10.017.
5. Zhou, X.; Xie, M.; Zhao, M.; Wang, Y.; Luo, J.; Lu, S.; Li, J.; Liu, Q. Pollution characteristics and human health risks of PM2.5-bound heavy metals: A 3-year observation in Suzhou, China. *Environ. Geochem. Health* **2023**, *45*, 5145–5162. https://doi.org/10.1007/s10653-023-01568-x.
6. Wei, X.; Liang, G.; Mao, J.; Yang, J.; Mu, Y.; Nong, C.; Zhang, D.; Lu, J.; Huang, X.; Wu, Y. Unraveling the main chemical mechanism of PM2.5 pollution caused by open burning of sugarcane straw during winter in southern China: The Pivotal Role of NOx and NH3 Oxidation-Neutralization. *Environ. Pollut.* **2025**, *381*, 126633.
7. Lee, J.H.; Yoshida, Y.; Turpin, B.J.; Hopke, P.K.; Poirot, R.L.; Lioy, P.J.; Oxley, J.C. Identification of Sources Contributing to Mid-Atlantic Regional Aerosol. *J. Air Waste Manag. Assoc.* **2002**, *52*, 1186–1205. https://doi.org/10.1080/10473289.2002.10470850.
